# JMJD6 and YBX1 physically interact and regulate HOTAIR proximal promoter

**DOI:** 10.1042/BCJ20243020

**Published:** 2025-09-02

**Authors:** Aritra Gupta, Siddharth Bhardwaj, Kartiki V. Desai

**Affiliations:** 1 Biotechnology Research Innovation Council-National Institute of Biomedical Genomics (BRIC-NIBMG), Kalyani, West Bengal, India; 2 Regional Centre for Biotechnology, Faridabad, Haryana, India

**Keywords:** breast cancer, co-immunoprecipitation, gene regulation, long intervening noncoding RNA

## Abstract

Earlier, we showed that jumonji domain containing protein 6 (JMJD6) interacted with HOTAIR promoter (−123 to −103 bp, termed JMJD6 interaction region [JIR]) and for maximal induction, an additional (−216 to −123 bp) region was required. *In silico* prediction and ENCODE data from MCF7 cells showed Y-box interacting protein 1 (YBX1) peaks in this region (YIR). Publicly available mass spectrometry data of proteins following JMJD6 immunoprecipitation identified YBX1 as an interacting partner. In this study, we validate JMJD6–YBX1 interaction in breast cancer cell lines using co-immunoprecipitation assays with recombinant, endogenous and *in vitro* synthesized proteins. Domain mapping using deletion constructs revealed that the A/P domain of YBX1 interacted with the JMJC domain of JMJD6. These proteins also positively regulated each other’s expression in breast cancer cell lines. Further, YBX1 augmented luciferase activity of HOTAIR promoter constructs, pHP216 and pHP123, in MCF7, Vec and JMJD6 overexpressing cells. siRNA-mediated depletion, mutation of YIR region or knocking out YBX1 (YKO cells) diminished luciferase activity. ChIP and ChIP-re-ChIP assays verified co-occupancy of both proteins in the HOTAIR promoter region. Electrophoretic mobility shift assays confirmed complex formation with YIR and JIR probes. Mutation of the YIR region and YKO resulted in loss of complex formation with both probes. Taken together, these data imply that YBX1 is crucial for physically recruiting JMJD6 to the HOTAIR promoter. Their interaction and positive feed-forward loop, perpetuated by JMJD6 and YBX1 inter-regulation, culminates in HOTAIR induction, which in turn is known to drive tumour progression.

## Introduction

Jumonji domain containing protein 6 (JMJD6) is an arginine demethylase and lysyl hydroxylase that epigenetically regulates gene transcription [1–4]. JMJD6 can modify transcription factors (TFs) to enhance or suppress their transcriptional activity. For example, while JMJD6 hydroxylates p53 negatively affecting its target gene transcription, it improves translocation of oestrogen receptor (ER) from the cytoplasm to nucleus. To achieve this, JMJD6 demethylates arginine residues on ER allowing ER transport [5,6]. In addition to translocating ER, JMJD6 is recruited to ER binding sites and is required for oestrogen-induced gene transcription in breast cancer cells [7]. JMJD6 has emerged as a key player in anti-pause release of stalled RNA pol II sites and augments gene transcription by bridging enhancer bound TFs and the basal transcriptional machinery [8].

We have earlier identified JMJD6 as a poor prognostic factor in breast cancer and shown its positive impact on cell proliferation and motility [9]. We recently showed that JMJD6 decreased ER expression and promoted insensitivity to endocrine therapy drugs such as Tamoxifen [10]. JMJD6 hijacked the E2-ER axis of proliferation by inducing E2F regulated genes and G2-M transitions to sustain breast cancer growth. Our previous data also indicates that JMJD6 and the long intervening non-coding RNA (lincRNA) HOTAIR are co-expressed in breast tumour samples, and their combined high expression promotes poorer survival in patients [11]. A JMJD6 interaction region (JIR) that positively regulated HOTAIR expression in breast cancer cell lines was also identified in the same study. JIR was found approximately -−123 to −103 bases upstream of the HOTAIR transcription start site (TSS). Addition of a further upstream 100 bp region (−216 to −123 bp) increased this basal promoter activity in JMJD6 overexpressing cells (JOE). Several potential transcription factor sites were predicted across this region but mutations of potential sites failed to identify the interacting protein(s). However, these regions displayed finite gel retardation complexes in the presence of nuclear extracts from JOE cells, suggesting the presence of other unidentified regulatory factor(s). To revisit potential interactors, we sampled two publicly available datasets. First, ENCODE ChIP data of MCF7 revealed potential binding sites for Y-box interacting protein 1 (YBX1) encompassing this region. Second, mass spectrometric data obtained from JMJD6 antibody pulldowns were further explored to identify potential interacting partners capable of DNA binding. This is because JMJD6 lacks a DNA-binding domain and relies on other DNA-binding factors for its recruitment to regulatory regions in the genome. Particularly, TFs including bromodomain containing protein 4 and MYC have been identified from such previously published datasets. Interestingly, YBX1 was also identified as a JMJD6 interacting protein in at least 3 published studies [4,8,12]. Further, YBX1 has functional similarities with JMJD6 such as association with poor prognosis, participation in mRNA splicing via U2AF factors, ability to bind RNA and decreasing ER expression [13–17]. Previous reports also show that HOTAIR in turn augments nuclear translocation of YBX1 and its oncogenic activity, raising the possibility of a regulatory loop between these proteins and lincRNA expression [18].

In the present paper, we combined ENCODE data and molecular studies to identify YBX1 as a possible factor that interacts with JMJD6 and the basal HOTAIR promoter. Here, we show that JMJD6 and YBX1 are expressed in a positive feed-forward loop, both proteins interact with each other physically and confirm that YBX1 interacts with the HOTAIR promoter to enhance its expression.

## Results

### *In silico* analysis of HOTAIR proximal promoter

**Figure 1:**
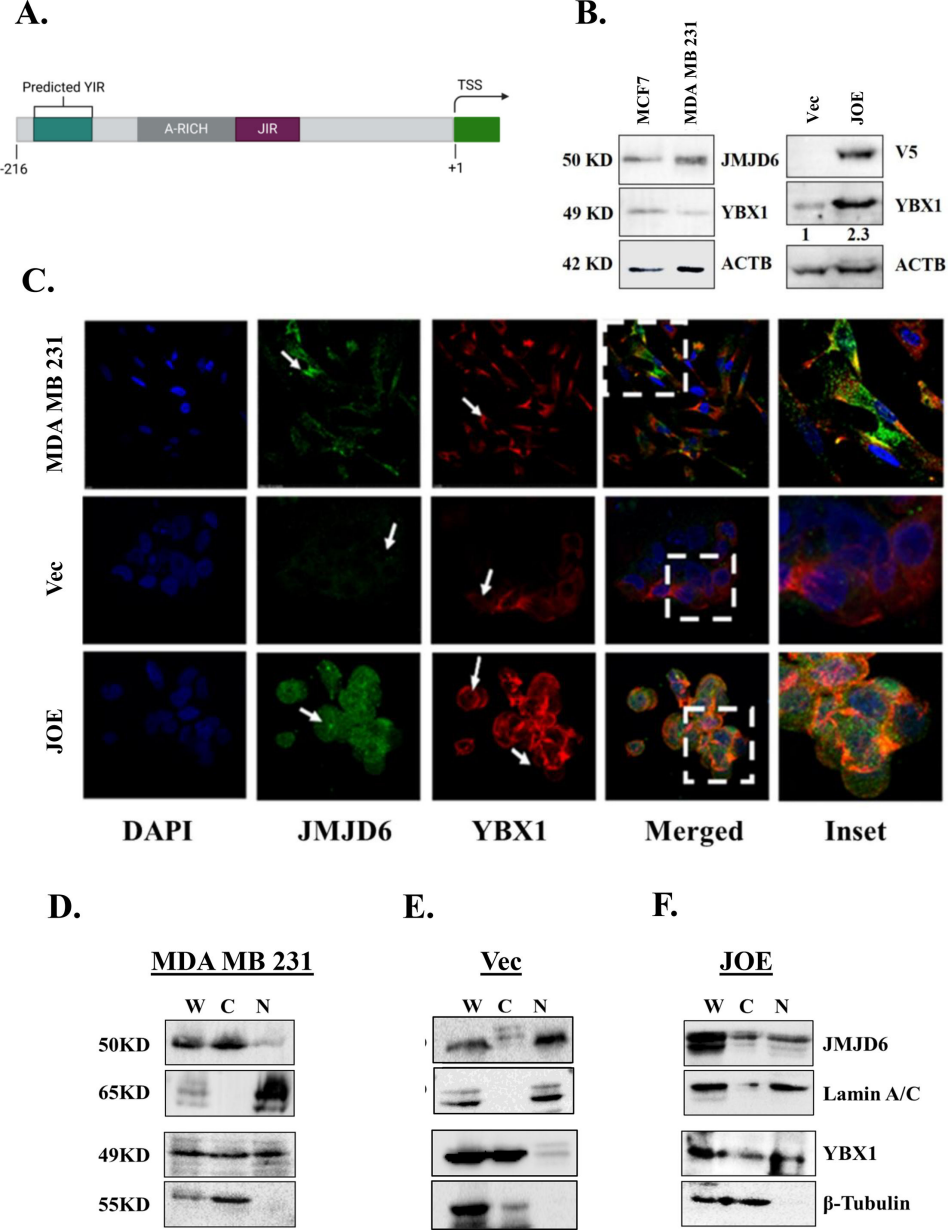
(**A**) Schematic representation of predicted YBX1 interaction region (YIR) in HOTAIR promoter; (**B**) western blot of JMJD6 and YBX1 in parental MCF7, MDA MB 231, Vec and JOE cells. Numbers below western panels from Vec and JOE cells indicate densitometric scan results of three independent experiments. Mean fold change in protein amounts between these two cell lines is shown. (**C**) Immunofluorescent detection of JMJD6 and YBX1 in MDA MB 231, Vec and JOE cells. Nuclear–cytoplasmic fractionation and immunoblots for JMJD6 and YBX1 in MDA MB 231 cells (**D**), Vec (**E**) and JOE (**F**) cells. C, cytoplasmic extract; *N,* nuclear extract; W, whole cell lysate.

Our previous data indicated that JIR (−123 to −103 bp) and an additional upstream region (−216 to −123 bp) of the HOTAIR promoter were required for JMJD6-mediated up-regulation. Observations from electromobility shift assay (EMSA) data using DNA probes spanning the (−216 to −123 bp) region raised the possibility that this upstream site may interact with additional transcription factor(s) [11]. However, despite mutating predicted TF-binding site(s) in this region, luc activity of promoter constructs remained higher and unaffected. These data suggested that factors other than those predicted by databases such as TRANSFAC could be responsible. Here, we utilized *in silico* data from the ENCODE consortium containing an experimental matrix of 160 TFs in MCF-7 cells. We found low enrichment profiles (1.5–2.5-fold) for four TFs: EP300, FOXA1, ZBTB11 and YBX1 in basal promoter region of HOTAIR. Of these, the former three factors could be eliminated, since our previous mutation study indicated that despite disrupting their cognate binding sites, the promoter activity remained unchanged. To ascertain the most likely sequence that YBX1 could interact with in this region, motifs reported using YBX1 ChIP data were consulted [17]. Sites having (5′-A/GCCATGG-3′), a YBX1 RNA consensus-binding site identified in SAGE data, were one possibility. Such a partial site (5′-CATGG-3′) was mapped downstream of the sites we had mutated earlier (Figure 1A). Therefore, this site was explored further as a potential YBX1 interacting region (YIR).

### Expression levels of YBX1

Since the HOTAIR promoter was previously characterized by us in JOE, Vec and MDA MB 231 cell lines, YBX1 expression levels were determined by western blotting and immunofluorescence in these three cell lines (Figure 1B and C). Immunoblot analysis of YBX1 showed abundant expression in MCF7 and MDA MB 231 cells (Figure 1B). Since MCF7 cells had lower JMJD6 expression levels, stable overexpression of V5 tagged JMJD6 was achieved (referred to as JOE cells, Figure 1B). Cells expressing cognate empty vector were termed as Vec cells. Interestingly, YBX1 expression was augmented in JOE cells, suggesting that JMJD6 may positively regulate its expression (Figure 1B, right hand panel). Since JMJD6 is a nuclear protein and YBX1 is found in both nucleus and cytoplasm, immunofluorescence using JMJD6 and YBX1 antibodies was carried out in MDA MB 231, JOE and Vec cells. JMJD6 was predominantly cytoplasmic and had marginal nuclear expression in MDA MB 231 cells (Figure 1C). This was surprising but documented earlier [19,20]. In JOE cells, JMJD6 was a nuclear protein, as has been described earlier. YBX1 was present in both nucleus and cytoplasm in all cell lines (Figure 1C). The observed distribution in various cell lines was confirmed by western blots using whole cell (W), nuclear (N) and cytoplasmic extracts (C). YBX1 was present in both the cellular compartments in all three cell lines and JMJD6 was predominantly nuclear in Vec/JOE but largely cytoplasmic in MDA MB 231 cells (Figure 1D–F). Lamin A/C was used as a nuclear protein marker and β Tubulin was blotted to indicate enrichment of cytoplasmic proteins in the immunoblots.

### JMJD6 and YBX1 inter-regulate each other

**Figure 2:**
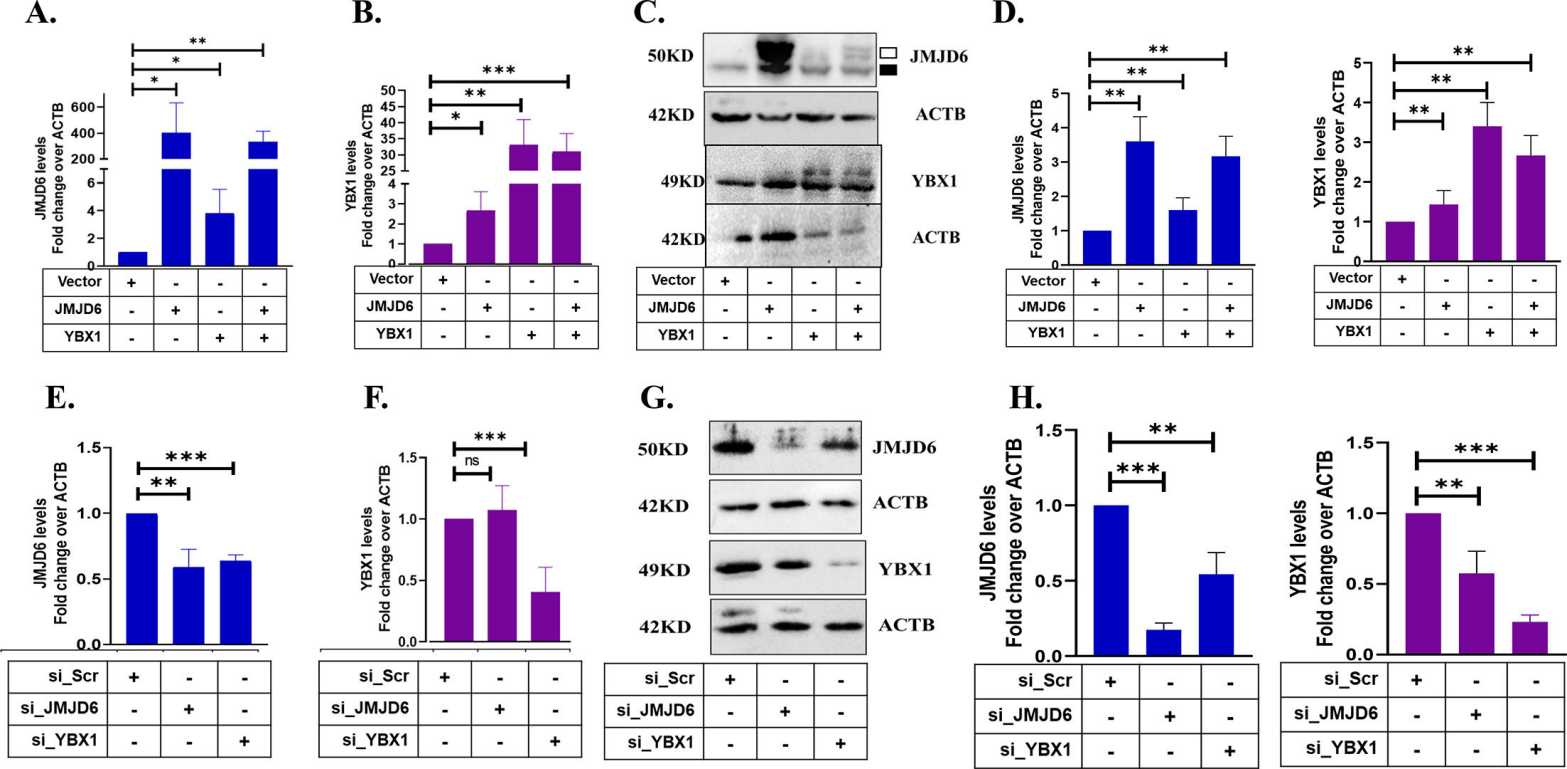
Expression levels of JMJD6 (**A**) and YBX1 (B) as quantified by quantitative RT-PCR following transient overexpression in MCF7 cells. (**C**) Western blots for both proteins in transfected cells. Endogenous JMJD6 is marked by filled square and empty square indicates V5-tagged protein. (**D**) Densitometric scans of western blots from three independent experiments. (**E**) and (F) Expression of JMJD6 and YBX1 following siRNA mediated knock down in MCF7 cells. Expression levels in scrambled siRNA (si_Scr) treated cells were normalized to 1. (**G**) Western blots for both proteins in siRNA-treated cells. (**H**) Densitometric scans of immunoblots from three independent siRNA treatments.

Interestingly, YBX1 expression was elevated in JOE cells suggesting that JMJD6 positively regulated YBX1, and we asked if the reverse was also true. Plasmids bearing JMJD6-V5 and Myc-YBX1 were transiently transfected in MCF7 cells and qRT-PCR analysis and immunoblots were examined for JMJD6 and YBX1 levels. YBX1 transfection induced both JMJD6 RNA and protein levels (Figure 2A and C) and JMJD6 induced YBX1 RNA and protein levels (Figure 2B and C). The densitometric scans of three independent experiments that quantify these western blots are shown in Figure 2D. In contrast, depletion of either JMJD6/YBX1 using gene-specific siRNAs resulted in down-regulation of JMJD6 RNA and protein levels (Figure 2E and G). Scrambled siRNAs (si_Scr) were used as control. On the other hand, YBX1 RNA levels were unaffected by JMJD6 depletion, though a decrease in protein levels was evident (Figure 2F and G). Figure 2H shows quantification of western blot data obtained from siRNA transfections. However, a decrease in protein amounts cannot be explained without invoking YBX1 mRNA stability and translatability. Further experiments are necessary to explain this observation. Overall, based on the protein levels, it appears that JMJD6 and YBX1 establish a feed-forward loop of expression that may augment their overlapping functions in cancer cells.

### JMJD6 and YBX1 interact with each other

**Figure 3:**
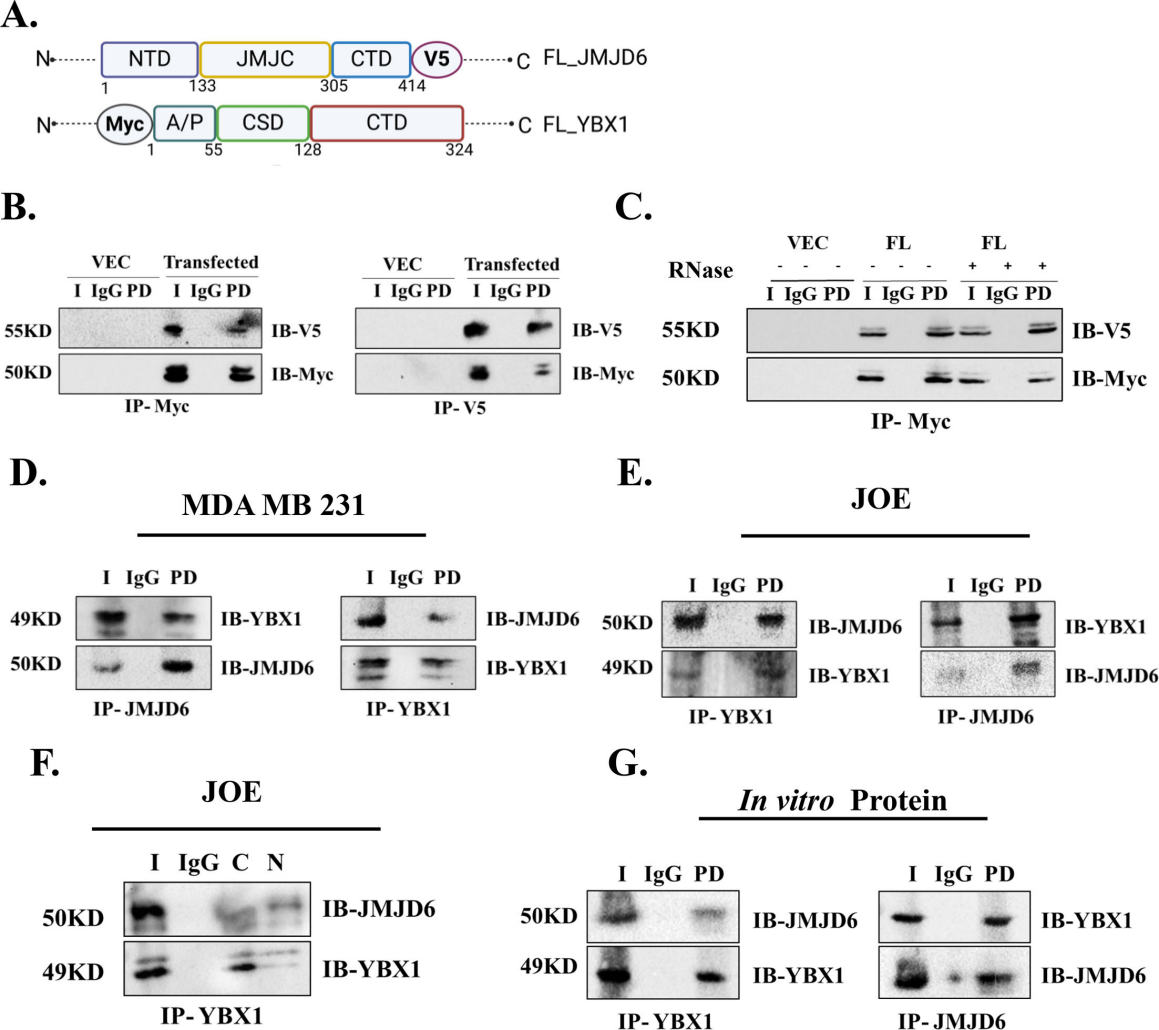
Interaction of JMJD6 and YBX1. (A) Schematic diagram of proteins used for recombinant expression in HEK293 cells. (**B**) CoIP using exogenously overexpressed proteins in HEK293 cells. (**C**) CoIP using recombinantly expressed proteins with/without RNase treatment in HEK293 cells. CoIP with endogenous proteins in MDA MB 231 (**D**) and JOE cells (**E**) . (**F**) CoIP in nuclear-cytoplasmic extracts of JOE cells. Input and IgG represent data from total cellular lysate, (**G**) CoIP with *in vitro* synthesized proteins. ‘I’ represents Input, ‘PD’ denotes immune precipitated protein, ‘IB’ represents antibody used for immunoblotting, and ‘IP’ denotes antibody used for pulldown.

To validate the mass spectrometry data, empty vector (Vec), or V5-tagged JMJD6 and Myc-tagged-YBX1 were co-expressed in HEK293 cells (Figure 3A). Co-IP was performed with V5-antibody (IP) and proteins obtained were assessed for Myc-antibody cross-reactivity to estimate YBX1 (IB) (Figure 3B). Similar assays were conducted using pull down with Myc and western blots with V5 antibody. Both co-IPs suggest that exogenously expressed proteins interacted with one another (Figure 3B). Since both JMJD6 and YBX1 are known to interact with RNA and could interact via this intermediate, the co-IP experiments were repeated in the presence and absence of RNAse in HEK293 cells. No difference was obtained under the two conditions, suggesting that RNA presence may not be essential for these proteins to interact (Figure 3C). Next, cell extracts bearing endogenously expressed proteins from MDA MB 231 and JOE cell lysates were subjected to immunoprecipitation with JMJD6 antibody and blotted for YBX1 and vice versa. Co-immunoprecipitation assays were successful under these conditions using total cell lysates (Figure 3D and E). Since Vec cells had negligible expression of JMJD6, co-IP experiments in these cells were not conducted. Similar co-IP experiments were conducted with nuclear (N) and cytoplasmic (C) lysates in JOE cells (Figure 3F). As shown, YBX1 IP co-precipitated JMJD6 in both nuclear and cytoplasmic compartments. In MDA MB231 cells, JMJD6 is predominantly expressed in the cytoplasm (Figure 1C). Accordingly, YBX1 pulldown detected JMJD6 only in the cytoplasm and not in the nucleus, probably due to suboptimal levels of nuclear expression (Supplementary Figure–S1A). These data demonstrate that JMJD6 and YBX1 interacted with one another in all cellular compartments when they were detectable under experimental conditions. To further demonstrate the interaction between these two proteins, they were synthesized using cell-free reticulocyte lysate systems and subjected to co-IP (Supplementary Figure–S1B,C). Figure 3G shows positive interaction of these *in vitro* synthesized proteins. In summary, across multiple cell lines, both proteins interact directly with each other when expressed recombinantly, or present endogenously or synthesized *in vitro*.

### Domain mapping of JMJD6 and YBX1

**Figure 4:**
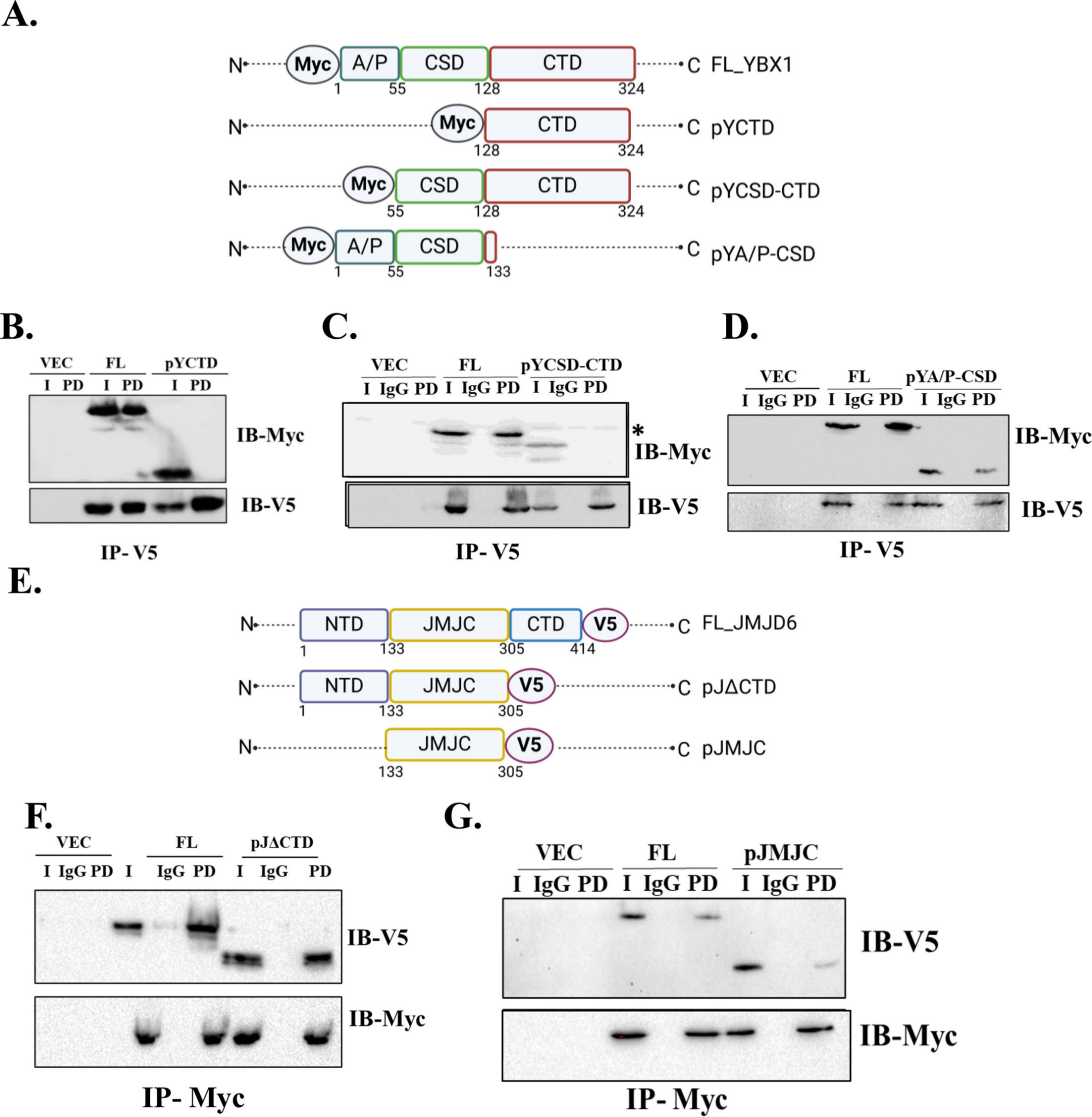
Domain mapping. (A) Myc- tagged deletion constructs of YBX1. CoIP of full length JMJD6 with (B) pYCTD, (**C**) pYCSD-CTD, (**D**) pYA/P-CSD. (**E**) Deletion constructs of JMJD6 tagged with V5. CoIP of full length YBX1 with F) pJΔCTD, and G) pJMJC (* denotes endogenous Myc)

Next, the regions of interaction between JMJD6 and YBX1 were mapped. Three deletion constructs of YBX1 all retaining the N-terminal Myc tag were constructed as follows: i) pYCTD (128–324 amino acids), ii) pYCSD-CTD (55–324 amino acids), iii) pYA/P-CSD (1–133 amino acids) and their expression was checked in HEK293 cells (Figure 4A, Supplementary Figure–2A-C). Each deletion construct was paired with full-length JMJD6-V5 in co-transfection experiments in HEK293 cells in three independent experiments. More than one blot was needed to accommodate all pairings. Since all pulldowns were conducted simultaneously for all constructs using the same antibody, control IgG samples were loaded with the full-length constructs and not on blots for each pairing. As shown in Figure 4(B–D), only the pYA/P-CSD construct showed a positive interaction with full length JMJD6. For JMJD6, two deletion constructs, namely pJΔCTD (1–305 amino acids) and pJMJC (133–305 amino acids), both with C-terminal V5 tag were constructed and their size and expression was confirmed by HEK293 transfection (Figure 4E, Supplementary Figure–2D and E ). Each construct was co-expressed with full length YBX1-Myc protein. Upon immunoprecipitation with Myc, both JMJD6 constructs were detected with V5 antibody. This denotes that JMJC domain of JMJD6 was sufficient to bind with YBX1 (Figure 4F and G). In conclusion, the domain mapping experiments show that the A/P domain of YBX1 was essential for interacting with the JMJC domain of JMJD6.

### YBX1 induces the HOTAIR promoter

**Figure 5:**
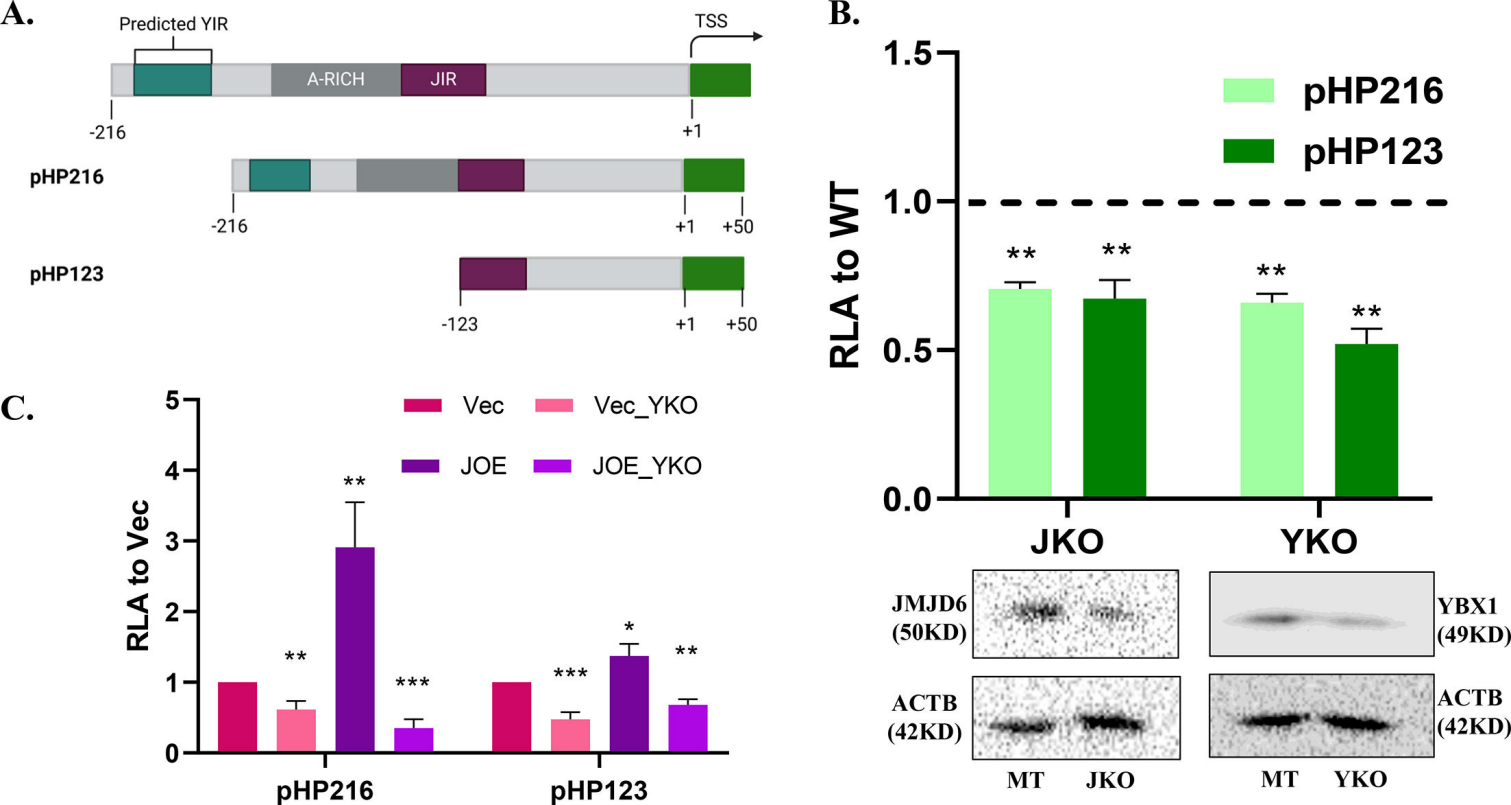
HOTAIR promoter regulation. (A) Details of HOTAIR promoter constructs. (**B**) Luciferase assays in MCF7 cells with transient knockout of JMJD6 (JKO) and YBX1(YKO). Dotted line represents luciferase activity in empty vector transfected cells normalized to 1. Extent of protein depletion is shown by western blots in the lower panel. (**C**) Luciferase assay in Vec and JOE cells after YBX1 KO. Vec cells were used as control and in each biological replicate RLA of constructs in Vec cells was normalized to 1. Fold change in activity was calculated over Vec cells.

We used two promoter constructs pHP216 and pHP123 to determine the effect of YBX1 expression on luciferase activity (Figure 5A). MCF7 cells were subjected to transient CRISPR-Cas9-mediated knock out of JMJD6 (JKO) and YBX1 (YKO), and pooled clones were used for pHP transfection. Figure 5B (upper panel) shows decreased promoter activity in JKO and YKO clones. Repeatedly, single clones of KO cells were not obtained due to limited growth or death of KO cells. Therefore, decrease in protein expression was used to confirm partial KOs by western blots prior to promoter assays (Figure 5B, lower panel). A 30–50% reduction in luc activity was evident when either of the proteins was depleted as compared with the parental MCF7 cells. These data show that both proteins were required to maintain HOTAIR promoter activity. Since HOTAIR had enhanced activity in JOE cells, we studied the effect of YBX1 KO on promoter constructs in JOE cells. Companion Vec cells were used as a control to normalize changes in luc activity. Strikingly, YBX1 affected both the basal and JMJD6-induced HOTAIR promoter activity (Figure 5C). However, as the YBX1 site was predicted in the (−216 bp to −123 bp) region, loss of pHP123 construct activity was unexpected. These data first suggested that YBX1 may be required for recruiting JMJD6 to its site in the pHP123 construct and that YBX1 was a more impactful regulator of the HOTAIR.

### YBX1 interacts with HOTAIR promoter

**Figure 6:**
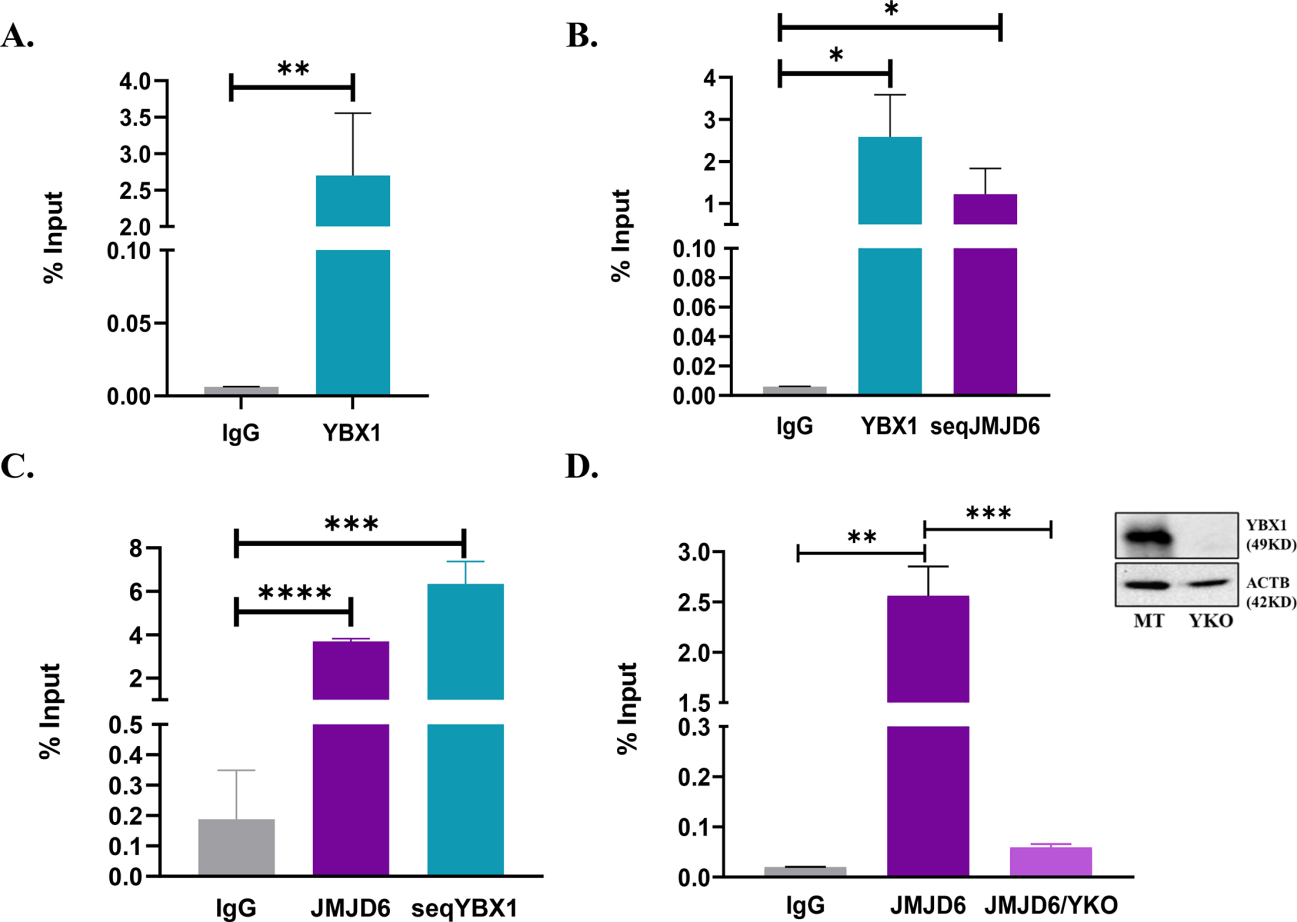
ChIP followed by sequential ChIP assays for HOTAIR promoter (−216 to + 50 bp) in JOE cells. (**A**) YBX1 ChIP. (**B**) YBX1 ChIP followed by JMJD6 pull down (seqJMJD6) and (C) vice versa. (**D**) JMJD6 ChIP in mock transfected (MT) and YKO cells. YBX1 protein levels are shown by western blot (inset).

Since YBX1 affected HOTAIR promoter activity, YBX1 ChIP was performed in JOE cells and the resultant DNA material was amplified with primers encompassing the 216 bp promoter proximal region of HOTAIR. YBX1 ChIP material was validated using previously reported binding sites in the SOX2A and MET1 regulatory regions (Supplementary Figure–S3) [21,22]. A substantial positive enrichment for YBX1 was observed in the proximal promoter region of HOTAIR (Figure 6A). Since the neighbouring region was identified by us as JIR, and both JMJD6 and YBX1 interact, we performed ChIP-re-ChIP assays in JOE cells to assess whether both proteins simultaneously occupy this region. We pulled down chromatin using YBX1 antibody followed by sequential JMJD6 pull down (seqJMJD6) and vice versa (Figure 6B and C). PCR data showed significant enrichment using ChIP and re-ChIP material in both cases. Both proteins, therefore, are likely occupying nearby regions in the promoter region of HOTAIR. To determine whether JMJD6 binding and/or activity was influenced by YBX1 binding close by, we performed YKO in JOE cells and chromatin from these cells was subjected to JMJD6 ChIP. (Figure 6D). The level of enrichment in YKO cells decreased substantially as compared with mock-transfected JOE cells (MT). These data recapitulate the data of the luc assays and reinforce the idea that YBX1 helps in JMJD6 recruitment at the JIR site.

### YBX1 interacts with (-216 to -123 bp) region

**Figure 7:**
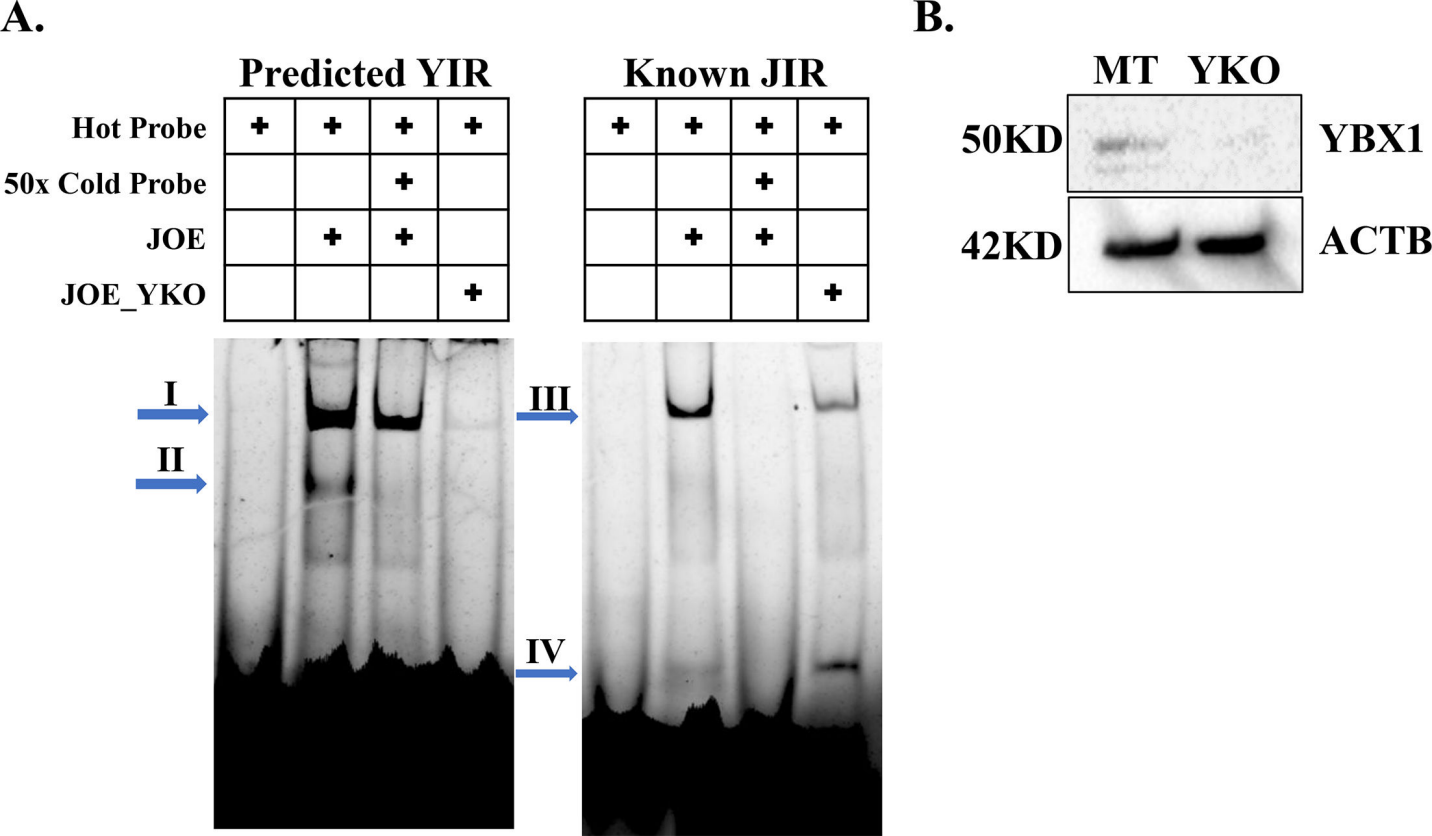
YBX1 interacting region of HOTAIR promoter. (**A**) EMSA of potential YIR and known JIR using nuclear extracts from JOE cells with and without competing cold probe and in the presence/absence of YBX1. Right-ward arrows denote complexes (**I-IV**). **B**) Western Blot to confirm depletion of YBX1 in JOE cells. MT, mock transfected cells.

The potential site of YBX1 interaction (YIR) was between −216 and −123 bp. We constructed two probes, one spanning this YIR and another to the known JIR (Supplementary Table–S1). EMSAs were performed using nuclear extracts from JOE and JOE_YKO cells. For potential YIR, two complexes appeared (complex I and II) and the 50X excess of cold probe removed complex II and reduced the intensity of Complex I (Figure 7A, left panel). Nuclear extracts from JOE_YKO cells completely abolished all complexes indicating that YBX1 was responsible for these shifts. As shown earlier, the JIR region retarded the cognate probe (complex III), 50X cold excess probe competed it out and loss of YBX1 (JOE_YKO) resulted in significantly weakening the intensity of the retarded complex. Appearance of complex IV was observed in the YKO lane; however, binding partner for this shift is unknown (Figure 7A, right panel). Immunoblot for mock transfected JOE (MT) and YKO cells is shown in Figure 7B. These data conclude that YBX1 interacts with the potential YIR and indicates that JMJD6 binding to JIR is probably depleted in the absence of YBX1.

### Mutation of the YIR region abrogates YBX1 binding

**Figure 8:**
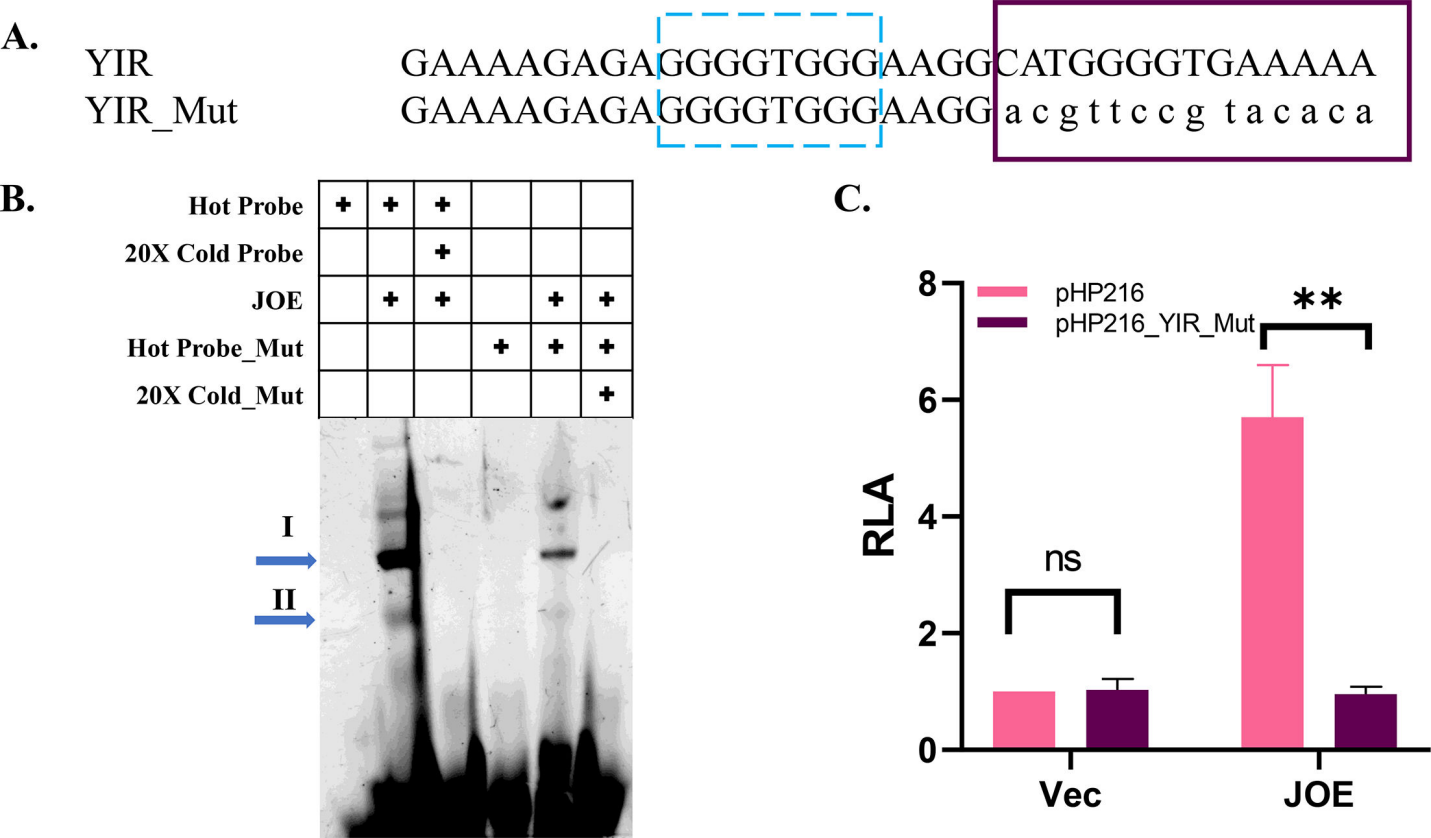
Mutation of the YIR binding site. (**A**) Schematic of mutations made in potential YBX1 binding site (pHP216_YIR_mut). Small case letters show the changes made from the wild type sequence. Dashed blue box indicates region mutated earlier [11] and magenta box represents predicted site. (**B**) EMSA with potential YIR and YIR_Mut probes using nuclear extracts from JOE cells, with and without competing cold probe. Right-ward arrows denote complexes (**I and II**).(**C**) Luciferase assay of pHP216 and pHP216_YIR_mut in Vec and JOE cells. Vec cells were used as control and in each biological replicate RLA of constructs in Vec cells was normalized to 1. Fold change in activity was calculated over Vec cells.

To verify that the potential YIR region was important for YBX1 binding and pHP216 luc activity, the predicted YBX1 binding site was mutated as shown in Figure 8A. Probes specific to YIR and YIR_Mut were subjected to EMSA using nuclear extracts from JOE cells. As observed earlier in JOE extracts, complex II was abolished and the intensity of complex I was compromised when YIR_Mut probe was used (Figure 8B). 20X molar excess of probes abolished all complex formation. pHP216 and pHP216_YIR_Mut constructs were transfected in Vec and JOE cells. Relative luciferase activity of pHP216_YIR_Mut in JOE cells was reduced to pHP216 levels observed in Vec cells, indicating that the JMJD6 mediated induction of HOTAIR was dependent on YBX1 binding to YIR (Figure 8C). However, the activity of the mutant promoter construct in Vec cells was maintained at a similar level to that of pHP216 level, indicating that this mutation did not affect the residual/basal activity but only subdued the JMJD6-related induction of HOTAIR.

### YBX1 positively regulates HOTAIR RNA expression

**Figure 9:**
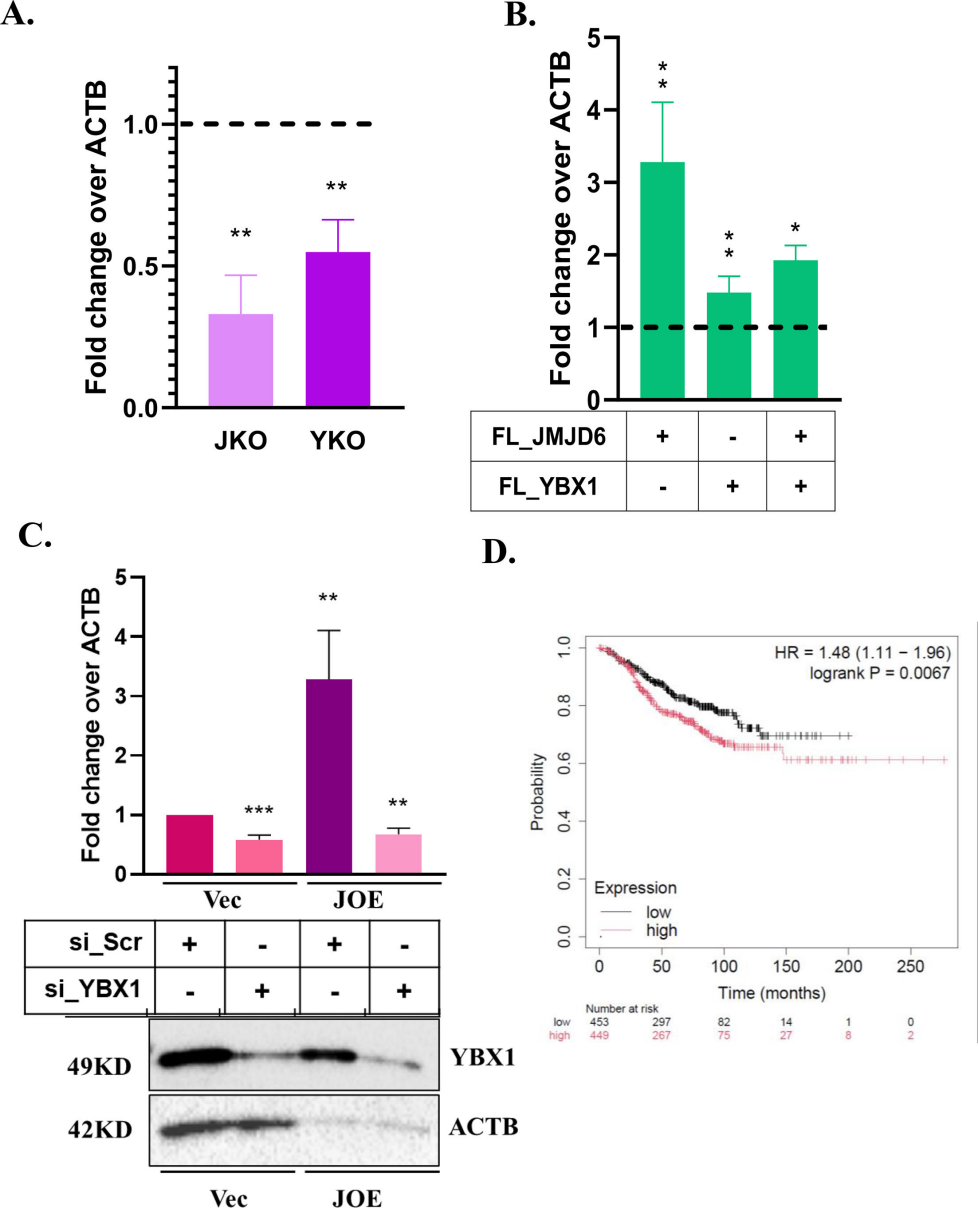
HOTAIR RNA expression. (**A**) qRT-PCR of HOTAIR in JKO and YKO cells. Dotted line represents HOTAIR levels in control MT cells, normalized to 1. (**B**) qRT-PCR of HOTAIR in MCF7 cells after transient overexpression of the two proteins. Expression levels of empty vector are marked as a dotted line and converted to 1. (**C**) qRT-PCR of HOTAIR in Vec and JOE cells following YBX1 siRNA mediated KD. HOTAIR levels in scrambled siRNA (si_Scr) were used as control and converted to 1. Western blots are shown in lower panel. (**D**) Survival analysis using all three genes (JMJD6, YBX1, HOTAIR), red line denotes high expression of all three and black line denotes low expression of all three genes. Lower panel represents patient number.

Quantitative real-time PCR assessments under various conditions of presence/absence of the two proteins were conducted to test if their binding results in change in HOTAIR expression. Depletion of either JMJD6 or YBX1 resulted in decreased HOTAIR RNA expression in MCF7 cells (Figure 9A). Transient overexpression of JMJD6 and YBX1 increased the level of HOTAIR RNA in MCF7 cells (Figure 9B). Similarly, siRNA mediated depletion of YBX1 in JOE and Vec cells significantly reduced HOTAIR expression in both cells (Figure 9C, upper panel). Therefore, YBX1 affects the basal levels of HOTAIR RNA and high JMJD6 alone is not sufficient to maintain the elevated HOTAIR levels. This observation further supports YBX1 mediated recruitment of JMJD6. To evaluate whether these observations in cell lines have any clinical relevance, publicly available data were studied. Tumours bearing high expression of all 3 genes in breast cancer were more prone to high hazard ratio and were associated with poorer survival (Figure 9D).

### Discussion

Earlier, we identified a JMJD6 inducible region (JIR) within 120 bases of the HOTAIR TSS and showed that addition of another 130 bases upstream (−216 to +50 region) doubled JMJD6 induced luc activity. However, we were unable to find the factor responsible for this improvement, although TRANSFAC predicted several proteins as potential interactors. Mutation of predicted binding regions failed to decrease the promoter activity [11]. In the current study, we revisited this problem and analysed ENCODE ChIP data from MCF7 cells. Only four factors displayed low enrichment in this region. Of these, our previous mutations encompassed EP300, ZBTB11 and FOXA1. The factor that remained unexplored was YBX1. YBX1 has potential for binding to DNA as well as RNA and it is known to regulate both gene transcription and translation [19,20]. The ENCODE data tracks for YBX1 spanning the HOTAIR proximal promoter region were explored in the IGV browser (data not shown), and a potential YBX1 interacting region was evident near the (−216 to −100 bp) region. This was approximately 60 bp upstream of JIR. In addition, previous studies identifying JMJD6 interacting proteins using JMJD6 IP listed YBX1 as one of the interacting partners [4,8]. These two observations prompted us to hypothesize that YBX1 physically interacts with JMJD6 and regulates the HOTAIR promoter by binding near JIR.

Immunoblotting showed ample expression of YBX1 in MCF7, Vec, JOE and MDA MB 231 cells. Surprisingly, although JMJD6 is a well-characterized nuclear protein, MDA MB 231 cells had higher expression of this protein in the cytoplasm and this has been described earlier [23,24]. YBX1 was present in both cytoplasm and nucleus in all cells tested and interestingly, JOE cells showed an induction in YBX1 expression. This prompted us to investigate if the two proteins inter-regulated each other’s expression. We tested whether transient expression of JMJD6/YBX1 or both in MCF7 cells altered RNA and protein levels of the other. Western blots and RT-PCR confirm JMJD6-mediated up-regulation of YBX1 and YBX1-mediated enhancement in JMJD6 RNA and protein levels. We note that co-transfection of both proteins did not show an additive nor synergistic effect. Overexpression of two proteins simultaneously led to less amounts of expressions of both constructs, possibly due to promoter squelching – that is suboptimal expression via competition for basal transcriptional machinery between two strong viral promoters when co-transfected in the same cells. Second, in transient transfection, cells may take up either construct individually and only a portion of cells may take up both constructs. Nevertheless, the induction was evident in co-transfected cells. In contrast, both JMJD6 and YBX1 siRNAs depleted levels of JMJD6 RNA and protein levels. However, JMJD6 siRNA showed no alteration in YBX1 levels, albeit the protein levels were down-regulated. One possibility could be that while JMJD6 siRNA prevents transcriptional induction of YBX1, it’s likely that YBX1 mRNA stability is maintained independent of JMJD6. YBX1 is not known to affect its own mRNA stability but YBX1 protein is degraded by several post-translational modifications (PTMs). Further experiments are required to find factors responsible for the reduction in YBX1 protein amounts.

Our investigations into published ChIP-seq data of JMJD6 and YBX1 did not reveal binding peaks for either proteins within regulatory regions of these genes. It is more likely that they are regulated by an independent TF. One such factor may be Myc. Myc up-regulates JMJD6 by binding to its promoter, and we and others have shown that JMJD6 induces MYC. Similarly, p73 recruits Myc-max complex to YBX1 promoter to enhance its expression [25]. In turn, the YBX1 and Myc axis is known to negatively affect cancer cell apoptosis, and YBX1 stabilizes Myc protein and allows enhanced Myc translation independent of the IRES when cells encounter stress [26]. These data suggest that a reciprocal and feed-forward regulatory loop may be established between JMJD6, YBX1 and MYC to enhance and sustain their cancer hallmark functions.

Although the inter-regulation was observed, the main aim of this paper was to determine whether JMJD6 and YBX1 interacted with one another and/or they were co-occupying the promoter region of HOTAIR to enhance its expression. Co-IPs were performed using JMJD6 or YBX1 antibodies followed by immunoblots to detect the interacting partner. Recombinantly overexpressed, *in vitro* synthesized and endogenous proteins consistently displayed positive protein-protein interaction in nuclear/cytoplasmic compartments in all cells tested in this manuscript. JMJD6 and YBX1 are both RNA binding proteins and to test whether this interaction involves RNA, co-IP was performed in presence or absence of RNase. The binding of these two proteins was agnostic to the presence of RNase and treatment did not hamper their interaction. These experiments suggest that direct interaction between these proteins is highly possible.

Next, we mapped protein domains involved in this interaction. YBX1 has three major domains: alanine proline rich domain (A/P domain), cold shock domain (CSD) and C terminal domain (CTD). Deletion constructs pYCTD and pYCSD-CTD were unable to bind full length JMJD6. This suggests that binding is probably within the A/P domain. The A/P domain is about 55 amino acids and finite expression and IP could not be achieved using this construct. Another JMJC family member, JARID2, also interacts with YBX1 [27]. This study successfully expressed A/P domain with 88 amino acids of CSD and similar clones were generated by us in this study [27]. This construct, pYA/P-CSD, could bind JMJD6. This indicates A/P domain of YBX1 was required for JMJD6 interaction. On the other hand, analysis of JMJD6 deletion constructs indicated that the JMJC domain could interact with pYA/P-CSD. We did not analyse any constructs of JMJD6 that were deleted for JMJD6 C-terminal domain since Cockman et al. have shown that mass spectrometric analysis using CTD truncated JMJD6 (beyond amino acid number 345) did not affect YBX1 pull down [12].

Using the Pepsite2 programme, we scanned 30 amino acid peptides generated over the pYA/P-CSD construct with +1 as the sliding window. The ‘QPPR’ amino acid sequence of the A/P domain displayed low and highly negative ΔG values. Whether JMJD6 enzymatically modifies YBX1 via arginine demethylation of the QPPR sequence would be an interesting aspect to study. Previously, removal of arginine methylation by JMJD6 from ER and G3BP1 (a stress granule protein) enhanced their nuclear localization [6]. JMJD6 also possesses a second enzymatic lysyl hydroxylase activity and several lysines in the disordered CTD of YBX1 are prone to modifications such as methylations, ubiquitinations, and so on. But in a recent study by Cockman et al., YBX1 was not identified as an enzymatic target of JMJD6 mediated lysyl hydroxylation [12]. More work in the future can be undertaken to explore if JMJD6 utilizes this strategy to concentrate YBX1 in the nucleus. Another PTM relevant to nuclear localization of YBX1 protein was its increased phosphorylation via ERK1, AKT or p70S6 kinase(s) [28]. Nuclear YBX1 promotes aggressive tumour behaviour and cellular invasiveness. Similarly, HOTAIR RNA binds YBX1 to translocate it to the nucleus and we have shown HOTAIR is induced by JMJD6 [18]. Phosphorylation of YBX1 induced multidrug resistance genes (such as MDR1) causing resistance to endocrine therapy drugs such as Tamoxifen. Similarly, high HOTAIR expression is also involved in drug resistance. We have previously shown that JOE cells are less sensitive to Tamoxifen and harbour high levels of phospho-ERK and receptor tyrosine kinase RET levels. Both contribute to drug resistance [29]. YBX1 was expressed in both nucleus and cytoplasm of MCF7 cells. It is plausible that JMJD6 induction of receptor tyrosine kinase RET and ERK1 contributes to enhanced YBX1 phosphorylation and translocation to the nucleus. Apart from the JMJD6-YBX1-Myc axis, the JMJD6-YBX1-HOTAIR axis may also prevail to promote cancer properties in tumours that possess high levels of these proteins and RNA. It is even more interesting to establish if, once nuclear, YBX1 in turn promotes HOTAIR induction, imposing additional advantage to invasive cancer cells.

Towards this, we tested whether YBX1 had any role in HOTAIR promoter regulation using the pHP216 (−216 to +50 bp) and pHP123 (−123 to +50 bp) constructs. In MCF7 cells, transient KO of either JMJD6 or YBX1 resulted in about a 30–50% loss of luc activity of both constructs. To estimate the contribution of YBX1 independently of JMJD6, we used Vec and JOE cells, since JMJD6 expression is marginal in MCF7, high in JOE but YBX1 is abundantly expressed in these cells. YBX1 expression was depleted in these cells by CRISPR-Cas9 mediated knock down. In both Vec_YKO and JOE_YKO cells, relative luciferase activity of pHP216 and pHP123 diminished. However, it was surprising that pHP123 luc activity decreased after YKO, since this construct lacked the potential YIR region present in pHP216. These observations, along with the co-IP interaction data, gave a first clue that YBX1 may recruit JMJD6 to the HOTAIR promoter. ChIP assays were then conducted and YBX1 specific ChIP DNA showed significant enrichment with HOTAIR promoter specific primers. To ascertain if both factors co-occupied the promoter region simultaneously, sequential ChIP-re-ChIP assays were performed using chromatin material from JMJD6 ChIP for re-CHIP using YBX1 antibody and vice-versa. Both ChIP-re-ChIP assays indicated enrichment for both proteins, suggesting they occupy neighbouring region(s) in the HOTAIR promoter. Interestingly, YKO resulted in a decrease in JMJD6 occupancy, confirming the dependency of JMJD6 on YBX1 presence in the promoter region. Next, we constructed two non-overlapping probes spanning the potential YIR and the known JIR. Gel-shift experiments identified 2 retarded complexes using nuclear extracts from JOE cells. Complex II could be competed out using 50X molar excess of cold probe, whereas a slight decrease in signal, if at best, was observed for complex I. Intriguingly, YKO in JOE cells completely abolished both complexes. This indicated that it was highly likely that YBX1 was at least one of the proteins that interacted with this region. More interesting were the complexes obtained when JIR probes were used in similar conditions. A single complex (III) was observed that was competed out by the cold probe. Surprisingly, YKO extracts decreased complex III formation, and another small complex (IV) was intensified. The appearance of such small complexes has been attributed to the presence of histones in several other studies. Overall, these data confirm that YBX1 assisted JMJD6 binding to JIR and enhanced JMJD6-mediated HOTAIR promoter activity. Previously, we had mutated predicted binding sites in the (–216 to –123) region but had not achieved a decrease in promoter activity. Here, we demonstrate that mutation of a potential YBX1 site resulted in loss of promoter activity. Further, YIR complexes were severely depleted when mutant probes were employed in gel-shift assays. These data confirm the binding of YBX1 and its requirement to enhance promoter activity.

The question remained whether the binding of YBX1 and JMJD6 resulted in an increase in HOTAIR RNA expression. In several ways, we perturbed expression of both proteins and confirmed that these proteins augment HOTAIR RNA expression. Particularly, in JOE cells, removal of YBX1 caused reduction in HOTAIR RNA, indicating it participates in JMJD6-mediated up-regulation. Interestingly, the YIR and JIR regions are separated by an A-rich region and we have previously shown that deletion of this region uncouples the requirement of JMJD6 for HOTAIR induction and results in very high activity of TATA-luc constructs. We and others have suggested that this could be explained by the interaction of repressor proteins such as IRF-1 with the A-rich motif [30]. We found that in YKO cells, the expression of the repressor IRF-1 was highly induced (data not shown). Therefore, it is possible that YBX1 leads to IRF1 depletion at the A-rich motif near its own binding site, now allowing recruitment of YBX1 bound JMJD6 to the HOTAIR promoter. Since this region is very proximal to the HOTAIR TSS, involvement of multiple other basal transcription factors cannot be ignored, and these have been discussed earlier in our previous manuscript [11]. Further studies are required to explore all the complexities involved in the regulation of HOTAIR.

Our data have shown that JMJD6 and YBX1 induce each other, bind each other and JMJD6 recruitment to HOTAIR promoter is dependent on YBX1 presence. Overall, these data emphatically suggests that JMJD6-YBX1-HOTAIR are engaged in a feed-forward positive loop. Once established, these three genes lead to aggressive tumour behaviour, poor response to therapy and ultimately poorer survival outcome in ER+patients. JMJD6 inhibitor has been used to regress tumours in preclinical mouse models and several efforts are being made to design YBX1 inhibitors since it promotes breast cancer metastasis [31,32]. Therefore, targeting either of these two proteins, or the interactive surfaces shared by these two proteins, may be a viable strategy to combat invasive and aggressive breast cancer.

## Materials and methods

### Cell culture

MCF7, MDA MB 231, HEK293 cells were purchased from American Type Culture Collection (ATCC, VA, U.S.A.). The cell lines were grown in Dulbecco’s Modified Eagle’s Medium (DMEM) (GIBCO, U.S.A.) with 5% fetal bovine serum (GIBCO, U.S.A.) and 1% Penicillin-streptomycin (GIBCO, U.S.A.) in humidified 5% CO2 incubator at 37°C. MCF7 cells stably expressing recombinant JMJD6 (referred as JOE cells) and control MCF7 cells carrying only the empty vector (Vec) have been described by us earlier [9]. Stable clones were maintained in 750 µg/ml Geneticin (Thermo Scientific, U.S.A.).

### Over expression of JMJD6 and YBX1

Construction of JMJD6-V5 plasmid was previously summarized and pDEST Myc (#19878) tagged YBX1 plasmid was purchased from Addgene [9]. PCR-based deletion constructs of JMJD6 and YBX1 were made using primers described in Supplementary Table–S1. Plasmids were transiently (co)-transfected in various cell lines using standard protocols and Lipofectamine 2000 (Thermo Scientific, U.S.A.) for various experiments detailed below.

### Depletion of JMJD6 and YBX1

SiRNA-mediated knock down (KD) of JMJD6 and YBX1 was performed using reverse transfection method. JMJD6 siRNA (5′GCUAUGGUGAACACCCUAATT 3′), YBX1 siRNA (5′ GUUCAAUGUAAGGAACGGAU3′) [9,33] were synthesized (Eurogentec) and for control non-targeting control (si_Scr) was used (Ambion). CRISPR based KO of JMJD6 and YBX1 was conducted. sgRNA with minimal off target effect was selected using CRISPOR tool and cloned in e-sp-Cas9-2a-GFP vector. Pooled KO clones were used for experiments since KO of JMJD6 and/or YBX1 proved to be lethal for cells in our hands.

### Western blots

Cells were lysed in RIPA buffer, with 1x Protease inhibitor cocktail (PI) (Sigma Aldrich) and protein extracts were quantified by the BCA method (Pierce, U.S.A.). Equal amounts (50 μg) of proteins were analysed in 10-15% SDS-PAGE gels, transferred on to PVDF membrane (Millipore, Germany) and nonspecific sites were blocked by using 5% non-fat milk (Bio-Rad, U.S.A.). Primary antibodies were incubated at 4°C overnight and after washing, blots were probed with suitable HRP-conjugated secondary antibody for 1 hour at room temperature. Signals were detected using HI-ECL (Bio-Rad, U.S.A.) in Chemidoc (Bio-Rad, U.S.A.). Antibodies used for Western blots were JMJD6 for total protein (endogenous and exogenous) (PSR-H7, Santacruz Biotechnology sc-28348, USA, 1:1000), V5 for exogenously expressed JMJD6-V5 (Thermo Scientific, U.S.A., R960-25, 1:5000), YBX1 (exogenous and endogenous) (Abcam, UK, ab-12148, 1:2000), Myc for exogenously expressed Myc-YBX1 (Sigma Aldrich, U.S.A., 05–419, 1:2000), β-Actin as internal control (Thermo Scientific, U.S.A., A2228, 1:5000), Lamin A/C (Abclonal, U.S.A., 1:5000, A0249) and β tubulin (Abclonal, U.S.A., 1:5000, AC008) for nuclear and cytoplasmic markers respectively. Densitometric scanning of three independent blots at variable exposures was conducted and data expressed as average numeric values under western blots wherever applicable.

### Immunofluorescence microscopy

A total of 5 × 10^4^ cells were plated onto poly-D-lysine-coated coverslips for 24 hours. Ten per cent neutral buffer formalin was used for fixation; after washing, cells were subjected to permeabilization (PBS + 0.2% Tween20) and a Glycine wash. Non-specific sites were blocked using 10% goat serum for 1 hour. Cells were incubated overnight with primary antibody, washed and incubated with fluorophore tagged secondary antibody for 1.5 hours (Abcam, UK). Coverslips were mounted using mounting media containing DAPI (Sigma Aldrich). Signals were captured in a confocal microscope (Nikon Eclipse, Ti2E).

### Cell fractionation

Cells were incubated with 5 X volume of cytoplasmic extraction buffer supplemented with PI and DTT for 3 minutes for isolating cytoplasmic protein fraction. The lysate was centrifuged and the supernatant reserved as the cytoplasmic fraction. The pellet was washed with cytoplasmic extraction buffer without NP40. After measuring packed nuclear volume, nuclei were lysed in nuclear extraction buffer containing 1X PI and DTT for 10 minutes. All processes were conducted at 4°C.

### Promoter reporter assays

pHP216_YBX1_Mut construct was derived from previously reported construct pHP216 containing (-216 to+50 bp) using overlap extension PCR with primers listed in Supplementary Table–S1. pHP123 plasmid carrying (−123 to +50 bp) region of HOTAIR promoter was described earlier [11]. All three constructs were used for assays in presence/absence of JMJD6/YBX1 or both. pRL-TK plasmid expressing Renilla luciferase under the control of thymidine kinase promoter was used as a control for determining transfection efficiency. Cells were transfected with various constructs using Lipofectamine 3000 and harvested 48 hours post transfection. Dual Luciferase Assay kit (Promega Corporation, WI, U.S.A.) was used to estimate luciferase activity. Relative luciferase activity was calculated by comparing with Firefly with Renilla luciferase activity. As applicable, empty vector or activity in Vec cells was used as control, and in each biological replicate, RLA of constructs in Vec cells was normalized to 1. Fold change in activity was calculated over Vec cells.

### Co-immunoprecipitation (co-IP)

A 500 µg of proteins from freshly prepared protein lysates of relevant cell lines was precleared using protein A/G agarose beads (Santacruz, U.S.A.). After nutation for 4 hours, the supernatant was mixed with antibody-bead slurry and kept overnight for nutation. Next day, after vigorous washing, proteins were eluted by boiling for 10 minutes in SDS-PAGE buffer in the presence of β-mercaptoethanol (BME). Immunoprecipitated complexes were detected by Western blots. 10% of extracts were reserved as input prior to co-IP. Veriblot (Abcam, UK) was used as a secondary antibody control.

### *In vitro* protein synthesis and interaction

JMJD6 and YBX1 constructs containing an upstream T7 promoter were linearized and used as a template for coupled transcription and translation in rabbit reticulocyte lysates according to manufacturer’s protocols (TnT kit, Promega Corporation, U.S.A.). Synthesis was confirmed using Western blot analysis using respective antibody. *In vitro* synthesized proteins were conjugated with respective antibody-A/G bead slurry. After 12 hours of nutation, non-specific proteins were washed off, followed by overnight nutation with the partner protein. Immunoprecipitated complexes were detected as described above.

### Chromatin immunoprecipitation (ChIP)

Cells were cross-linked using formaldehyde solution and the reaction was stopped using glycine. For JMJD6 ChIP, Di succinimidyl glutarate (DSG) was additionally used as a cross-linker. For JMJD6 ChIP, V5 antibody and for YBX1, YBX1 antibody was used. Chromatin isolated was sheared to 300 to 700 bp fragments using a bath sonicator (Covaris S200). Fragment size was confirmed by D1000 screen tape on Agilent tape station. Antibody pull down was performed overnight with A/G magnetic beads, followed by standard washing and protein DNA de-cross-linking. For sequential ChIP, ChIP material from the first pull down was not de-cross-linked; instead, incubation with the partner protein antibody was continued. Isolated DNA was run through PCR purification columns before quantitative PCR with primers for selected chromatin regions (Supplementary Table–S1). 10% input DNA was purified prior to ChIP and used for enrichment analysis using the Percentage Input method.

### Electromobility shift assay (EMSA)

Nuclear extracts were freshly prepared from 10^7^ cells. Cyanine-5 fluorescently labelled DNA probes (SIGMA chemical Co.) have been described earlier (Supplementary Table–S1) and the LUEGO protocol used for EMSA [34]. 100 nM probes were incubated with 10 µg of nuclear extracts in a binding buffer (10 mM Tris–HCl, pH7.6, 50 mM KCL, 1 mM DTT, 10% glycerol, 200 ng/reaction poly(dI-dC)) for 30 min on ice. Reaction mixtures were loaded onto a non-denaturing 5% acrylamide: bisacrylamide (29:1) gel and run for 45 min at 100 V in 0.5 × TBE buffer. 20–50X unlabelled cold competitor probe was used to determine specificity of complexes found. Gels were scanned after electrophoresis using ChemiDoc (Bio-Rad) using the Cy-5 channel. Extracts from pooled CRISPR KO clones were used as YKO protein samples.

### RNA isolation and quantitative RT-PCR (qRT-PCR)

RNA was isolated using RNAeasy isolation kit (Qiagen Corp.). 1 µg of RNA was taken for reverse transcription using random hexamer and SuperScript III (Thermo Scientific, USA). 1 µl of cDNA was used to perform qRT-PCR in thermal cycler (Bio-Rad) using specific primers (Supplementary Table–S1).

### Survival analysis

Breast cancer data from KM plotter ( www.kmplot.com/analysis) were utilized. For each gene, tumours expressing above median expression were grouped as high expressors and those below as low expressors. High expressors from all three genes were compared with tumours classified as low expressors for all three genes and probability of survival was estimated.

### Statistical analysis

All the experiments are done three times; graphs are made by GraphPad Prism software. Statistical analysis was done using Bioconductor R. Comparisons between two groups were performed using student’s paired t-test and data was considered statistically significant at *P*≤0.05. Significance in all figures is indicated as follows: * ≤ 0.05, ** ≤ 0.01, ***P ≤ 0.001 and ****P ≤ 0.0001.

## Supplementary material

Online supplementary figure 1

Online supplementary figure 2

Online supplementary figure 3

Online supplementary table 1

Uncited online supplementary material 1

## Data Availability

This manuscript does not have structural/crystallographic data for macromolecular structures and/or small molecules, nor protein and nucleic acid sequence data (including RNA Seq data), nor functional genomics and molecular interactions/proteomics/metabolomics data that requires deposition in repositories. No computational models or Genetics data (genetic polymorphisms; genotype data) were generated in this study. Therefore, data sharing is not applicable to the paper.

## Abbreviations

JIR: JMJD6 interaction region
JMJD6: jumonji domain containing protein 6
JOE: JMJD6 overexpressing cells
KO: knockout
TF: transcription factor
