# Supplementary figures and images for: JMJD6 and YBX1 physically interact and regulate HOTAIR proximal promoter
Source: Biochem J. 2025 Sep 2;482(17):1289–305. doi: 10.1042/BCJ20243020 (PMC12794343; doi:10.1042/BCJ20243020)

Supplementary Figure 1

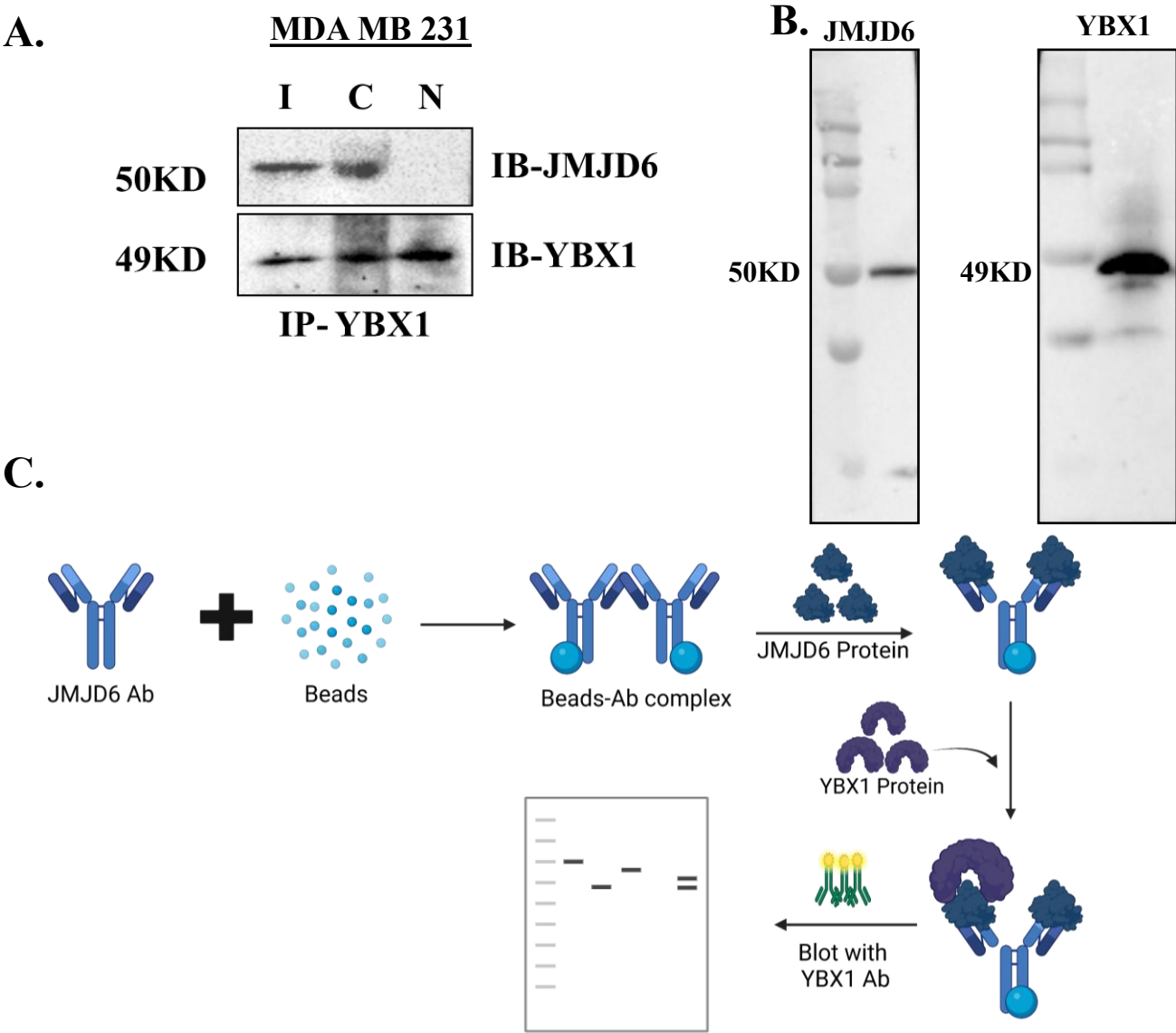

Supplement: Online supplementary figure 1 [file bcj-482-17-BCJ20243020-s001.pdf]

**Supplementary Figure 2**

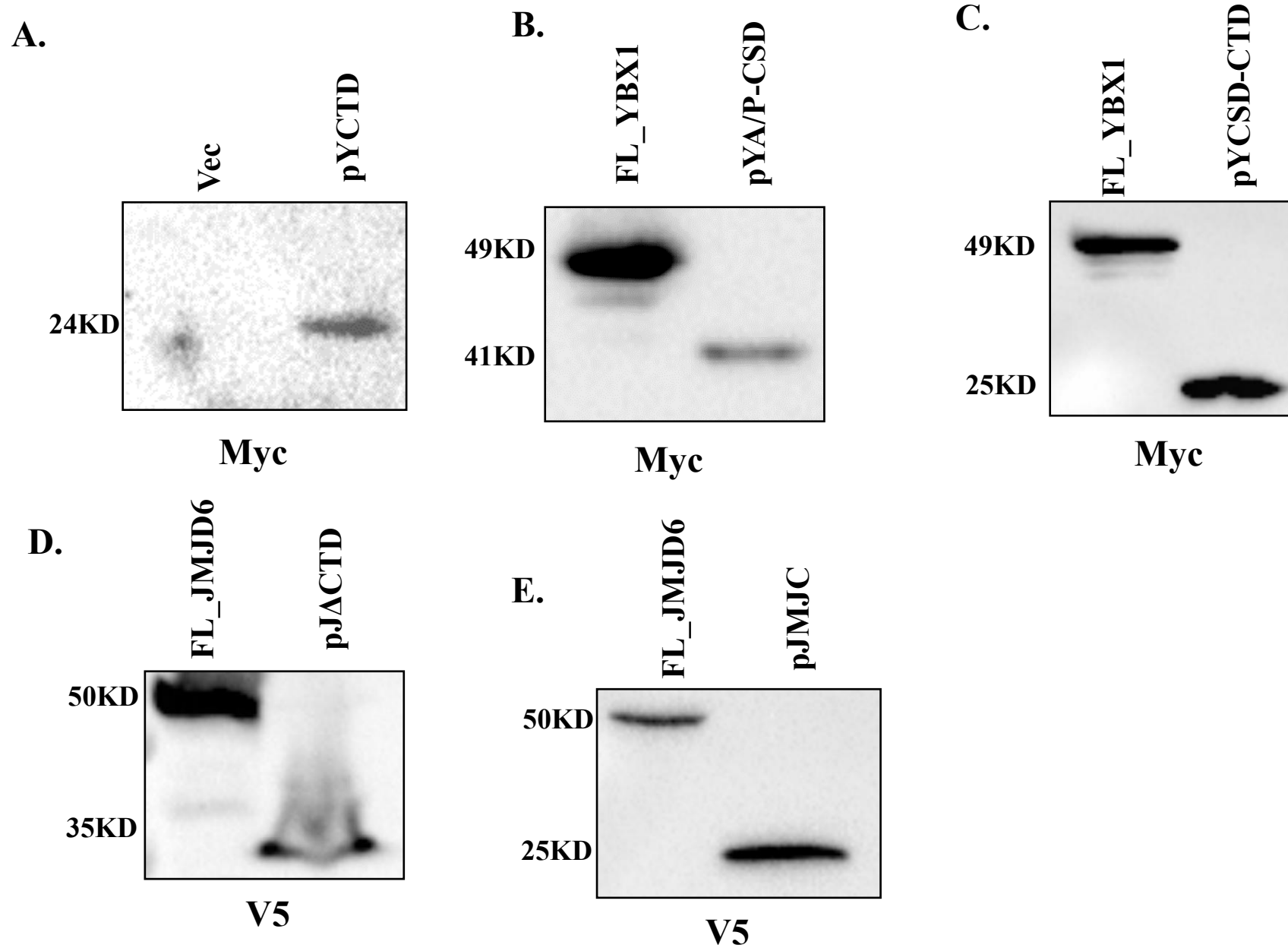

Supplement: Online supplementary figure 2 [file bcj-482-17-BCJ20243020-s002.pdf]

Supplementary Figure 3

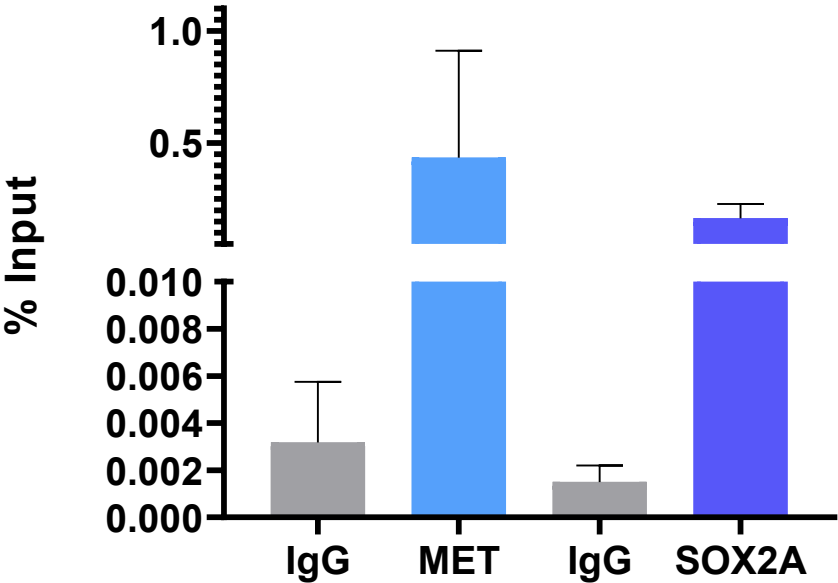

Supplement: Online supplementary figure 3 [file bcj-482-17-BCJ20243020-s003.pdf]
