# Supplementary material for: JMJD6 and YBX1 physically interact and regulate HOTAIR proximal promoter
Source: Biochem J. 2025 Sep 2;482(17):1289–305. doi: 10.1042/BCJ20243020 (PMC12794343; doi:10.1042/BCJ20243020)
Supplement: Uncited online supplementary material 1 [file bcj-482-17-BCJ20243020-s005.docx]

**Supplementary figures**

Supplementary figure 1: (A) CoIP of nuclear and cytoplasmic extracts of MDA MB 231 cells. B) Western blot of *in vitro* synthesised JMJD6 and YBX1 protein. C) Experimental strategy for CoIP with *in vitro* synthesized proteins.

Supplementary figure 2: (A-C) Western blots showing expression of various YBX1 deletion constructs following transfection in HEK293 cells. D) and E) Western blot of JMJD6 deletion constructs following transfection in HEK293 cells

Supplementary figure 3: Standardization of YBX1 ChIP using previously defined binding sites

**Supplementary Table**

Supplementary table 1: Oligo sequences
